# WHEN SHOULD BE CONVERTED LAPAROSCOPIC SLEEVE GASTRECTOMY TO LAPAROSCOPIC ROUX-EN-Y GASTRIC BYPASS DUE TO GASTROESOPHAGEAL REFLUX?

**DOI:** 10.1590/0102-672020200004e1553

**Published:** 2021-01-25

**Authors:** Italo BRAGHETTO, Owen KORN, Anamaría BURGOS, Manuel FIGUEROA

**Affiliations:** 1 Department of Surgery, University Hospital “Dr José J. Aguirre”, Faculty of Medicine, University of Chile, Santiago, Chile

**Keywords:** Sleeve gastrectomy, Gastroesophageal reflux, Gastric bypass, Gastrectomia vertical, Refluxo gastroesofágico, Bypass gástrico

## Abstract

***Background*:**

Gastroesophageal reflux (GER) is one of the most common indications for conversion of sleeve gastrectomy (LSG) to laparoscopic Roux-en-Y gastric bypass (LRYGBP). Objective evaluations are necessary in order to choose the best definitive treatment for these patients.

***Aim*:**

To present and describe the findings of the objective studies for gastroesophageal reflux disease performed before LSG conversion to LRYGBP in order to support the indication for surgery.

***Method*:**

Thirty-nine non-responder patients to proton pump inhibitors treatment after LSG were included in this prospective study. They did not present GER symptoms, esophagitis or hiatal hernia before LSG. Endoscopy, radiology, manometry, 24 h pH monitoring were performed.

***Results*:**

The mean time of appearance of reflux symptoms was 26.8+24.08 months (8-71). Erosive esophagitis was found in 33/39 symptomatic patients (84.6%) and Barrett´s esophagus in five. (12.8%). Manometry and acid reflux test were performed in 38/39 patients. Defective lower esophageal sphincter function was observed independent the grade of esophagitis or Barrett´s esophagus. Pathologic acid reflux with elevated DeMeester´s scores and % of time pH<4 was detected in all these patients. more significant in those with severe esophagitis and Barrett´s esophagus. Radiologic sleeve abnormalities were observed in 35 patients, mainly cardia dilatation (n=18) and hiatal hernia (n=11). Middle gastric stricture was observed in only six patients.

***Conclusion*:**

Patients with reflux symptoms and esophagitis or Barrett´s esophagus after SG present defective lower esophageal sphincter function and increased acid reflux. These conditions support the indication of conversion to LRYGBP.

## RESUMO

***Racional*:**

O refluxo gastroesofágico é uma das indicações mais comuns para a conversão da gastrectomia vertical (SG) em gastroplastia laparoscópica em Y-de-Roux (LRYGBP). Avaliações objetivas são necessárias para escolher o melhor tratamento definitivo para esses pacientes.

***Objetivo*:**

Apresentar e descrever os achados objetivos da doença do refluxo gastroesofágico realizados antes da conversão do SG para o LRYGBP, a fim de apoiar a indicação cirúrgica.

***Método*:**

Trinta e nove pacientes não respondedores ao tratamento com inibidores da bomba de prótons após SG foram incluídos neste estudo prospectivo. Eles não apresentavam sintomas de refluxo gastroesofágico, esofagite ou hérnia hiatal antes da SG. Endoscopia, radiologia, manometria, monitoramento de pH 24 horas foram realizados.

***Resultados*:**

O tempo médio de aparecimento dos sintomas de refluxo foi de 26,8+24,08 meses (8-71). Esofagite erosiva foi encontrada em 33/39 pacientes sintomáticos (84,6%) e esôfago de Barrett em cinco (12,8%). A manometria e o teste de refluxo ácido foram realizados em 38/39 pacientes. A função alterada do esfíncter inferior do esôfago foi observada independentemente do grau de esofagite ou esôfago de Barrett. Em todos esses pacientes, foi detectado refluxo ácido patológico com escores elevados de DeMeester e % de tempo pH<4, mais significativo nos com esofagite grave e esôfago de Barrett. Anormalidades radiológicas SG foram observadas em 35 pacientes, principalmente dilatação da cárdia (n=18) e hérnia hiatal (n=11). Estenose gástrica foi observada em apenas seis pacientes***.***

***Conclusão*:**

Pacientes com sintomas de refluxo e esofagite ou esôfago de Barrett após SG apresentam função do esfíncter esofágico inferior defeituosa e aumento do refluxo ácido. Esses sintomas e estudos objetivos apoiam a indicação de conversão para LRYGBP.

## INTRODUCTION

Laparoscopic sleeve gastrectomy (LSG) has emerged over the last few years as an ideal bariatric procedure because it has several advantages compared to more complex bariatric procedures. It is currently one of the most frequently performed bariatric interventions worldwide^8^ ^,^ ^16^. However, gastroesophageal reflux (GER) symptoms have been described after LSG^2^. This complication is one of the most common indications for conversion of LSG to laparoscopic Roux-en-Y gastric bypass (LRYGBP)^3^. It is important to evaluate the competence of lower esophageal sphincter (LES) and magnitude of acid reflux because they are the factors involved in the pathogenesis of GER symptoms and esophagitis after LSG. Therefore, this information is relevant for bariatric surgeons in order to establish the possible outcome and to choose the best treatment of these patients: either permanent treatment with PPIs or indicated revisional surgery. Up to now, there is no previous data concerning these measurements in patients converted to gastric bypass after SG, only clinical and endoscopic studies^3^ ^,^ ^8^ ^,^ ^14^ ^,^ ^16^. 

The aim of this study was to present and describe the findings of the objective studies for gastroesophageal reflux disease performed before LSG conversion to LRYGBP in order to support the indication for surgery. 

## METHOD

### Ethical disclosures

All procedures were in accordance with the Institution and Ministerial Committee and with the 1961 Helsinki declaration and its later amendments or comparable ethical standards. **T**he authors declare that they have followed the protocols of their work center on the publication of patient data and that no patient data appears in this article. Written informed consent was obtained from all individual participants included in the study. 

### Patients

In our team, all obese candidates to bariatric surgery are evaluated before the operation according to the specific protocol in order to precise the obesity index, presence of co-morbidities, clinical and psychological interview, nutritional evaluation, metabolic tests, abdominal ultrasound and upper gastrointestinal endoscopy. Patients without reflux symptoms, endoscopic esophagitis, presence of hiatal hernia or Barrett´s esophagus are eligible for SG (inclusion criteria). On the contrary, patients with GER symptoms and preoperative endoscopic esophagitis, Barrett´s esophagus or hiatal hernia were excluded (exclusion criteria). After discharge, according to our outcome protocol, patients received PPI medication for 1 or 2 months after sleeve. This therapy was stopped if they did not present reflux symptoms. In patients with them, appearing after the initial SG, were submitted prospectively to objective re-evaluations with manometry and 24 h pH monitoring endoscopic and radiologic examinations (inclusion criteria). 

**FIGURE 1:**
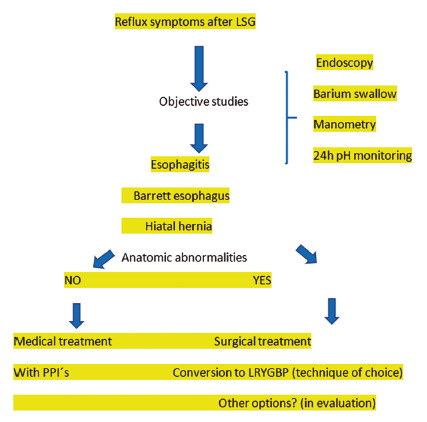
Patients and work-up before and after SG pre conversion to LRYGBP

In the last 11 years 658 patients were submitted to SG, with preoperative mean weight of 106+14.1 kg and mean BMI 38.4+13.4 kg/m^2^ (in our unit patients with BMI more than 45 are submitted to gastric bypass). The technique used is according to the one previously described^4^. The initial LSG was performed in a mean time of 5.6+2.5 years (2-11) before; 315 out of total (48%) had a complete and periodic follow-up by the multidisciplinary team, monthly during the first six months and annually after surgery (2-11 y); 205 patients out of 315 patients (65.1%) presented reflux symptoms during the follow-up and they received PPI´s treatment, among them, 39 patients, five men and 34 women with a mean age of 43.7+8.5 years (23-61) presented intractable reflux symptoms and were submitted to revaluation with manometry, upper gastrointestinal endoscopy and barium swallow radiologic study (inclusion criteria ) Figure 1.

### Symptoms

The presence and severity of typical reflux symptoms (heartburn or regurgitation) were recorded and classified using the modified DeMeester reflux symptoms score^28^.

### Endoscopy

Was performed in order to confirm or exclude the presence of reflux esophagitis which was classified according to Los Angeles´s classification^11^. Erosive esophagitis was found in 57 patients (27.8%), (41 with grade A, 11 grade B and five grade C). Barrett´s esophagus was observed in 10 patients. Hernia hiatal was defined when a part of the stomach slips up or passes (herniates) through the hiatus and into the chest. This condition was determined by endoscopic and radiologic assessment if the esophagogastric junction was located at least 2-3 cm above the hiatus with gastric folds ascending to this level. Also, manometry can determine the presence of hiatal hernia when lower esophageal sphincter is displaced 2-3 cm up to the hiatus^11^ ^,^ ^29^.

### Radiology

Radiological evaluation with a barium swallow examination and computed tomography acquisition was performed in order to detect sleeve modifications. Dilated cardia was diagnosed if the cardia diameter was more than 2.5 cm, and hiatal hernia if a segment of gastric fundus more than 2 cm above the crura was detected^20^ ^,^ ^29^.

### Esophageal functional studies

They were performed in 38/39 patients in order to evaluate the LES function and confirm acid reflux. Standard manometry or high definition manometry and 24 h pH monitoring were performed according to the methodology and equipment routinely used for this study^7^ ^,^ ^19^. 

### Statistical analysis

Chi-square, Fisher test and student ¨t¨ test were used according to the characteristics and distribution of the variables. For multiple mean an analysis of variances (ANOVA) was conducted to determine whether a statistically significant difference was present among the groups with a confidence of 95% (p<0.05). In case a difference existed, a Tukey Honest Statistical Difference (HSD) for Post-Hoc Analysis was performed to determine between which groups the difference was present. Statistical significance in this case was stablished when the difference between group means was higher than the HSD stablished for each variable. 

## RESULTS

In the 39 patients included the BMI post-LSG was 32.6+2.1 kg/m^2^. *Co*ncomitant weight regain after sleeve was observed in 23.6% of patients (mean=19.8+15.7 kg). The mean time of appearance of reflux symptoms after surgery was 26.8+24.1 months (8-71) and they received PPI´s treatment for at least six months, if persistence of symptoms was confirmed they were re-evaluated. After their complete re-evaluation the second operation was indicated. The interval between the first and the second operation was 4.6+2.41 years (2-10).

Postoperative complications occurred in four patients, two with Clavien-Dindo grade II (the first one presented internal bleeding at jejunojejunal anastomosis and the second one presented wound infection at trocar site and two with grade IIIa complications (intestinal obstruction due to port hernia and the other one presented mesenteric vein thrombosis). No conversion to open surgery or mortality was observed. 

Table 1 summarizes the clinical characteristic before and after SG demonstrating the findings of the objective studies before conversion to LRYGBP.

**TABLE 1:** Clinical characteristic of patients before and after sleeve gastrectomy before conversion to LRYGBP in 315 patients with complete follow-up

Before sleeve gastrectomy		After sleeve gastrectomy	
GER symptoms	Absent*	Yes	205 patients (65.1%)
PPI´s treatment	Absent	Yes	205 patients (65.1 %)
Non-responder			39 patients (12.3%)
Endoscopic esophagitis	Absent	Yes	57 patients (27.8 %)
41 Grade A			
11 Grade B			
5 Grade C			
Barrett´s esophagus	Absent	Yes	10/315 entire group (3.2%)
			10/205 symptomatic patients (4.8%)
			10/39 non-responder to PPI´s (25.7%)
Hiatal hernia	Absent	Yes	11/315 entire group (3.4%)
			11/205 symptomatic patients (5.3%)
Cardia dilatation	Absent	Yes	18/205 symptomatic patients (8.7%)
Manometry#	N/E**	LES incompetence	38/39 patients (97.4%)
24h pH monitoring#	N/E**	Abnormal acid reflux	38/39 patients (97.4%)

*Absent=patients without reflux symptoms;PPI´s=treatment or esophagitis before SG; **N/E=not evaluated; LES=lower esophageal sphincter; #=manometry and pH monitoring in 38/39 patients

**FIGURE 2:**
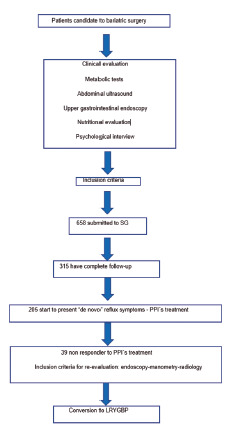
Endoscopic evaluation: A) esophagitis grade B after sleeve gastrectomy with small hiatal hernia; B) paraesophageal hiatal hernia, “U turn”; C) mesogastric stricture before conversion to LRYGBP

In Table 2 the severity of reflux symptoms and endoscopic findings are presented. Esophagitis grade A was the most frequent finding, however more symptomatic patients presented more severe damage of mucosa (esophagitis grade C or Barrett´s esophagus), compared to the other groups, but not significant (p=0.34, Figure 2).

**TABLE 2:** Reflux symptoms and endoscopic findings after LSG in patients who were converted to LRYGBP.

Endoscopic esophagitis						
Symptoms	n	No n (%)	Grade A n (%)	Grade B n (%)	Grade C n (%)	Barrett´s esophagusn (%)
Mild	9	1	5 (55.5)	1 (11.1)	1 (11.1)	1 (11.1)
Moderate	8	4 (50)	4 (50)			
Severe	22	6 (27.3)	8 (36.4)	4 (18.2)	4 (18.2)*	
Total	39	1	15 (38.5)	13 (33.3)	5 (12.8)	5 (12.8)

*No statistically significant differences between groups (p=0.34)

In Table 3 manometric characteristics of the LES and 24 h pH monitoring in 38 patients with erosive esophagitis performed before conversion to LRYGBP are presented. Defective LES was observed in these 38 patients. Hypotensive LES was detected in all patients with LES pressure below 7mmHg and total length of the sphincter was less than 4 cm, without differences among the patients with esophagitis or Barrett´s esophagus. (p=0.58). Intra-abdominal sphincter was not observed in most patients. 

**TABLE 3:** Manometry, 24 h Ph monitoring and endoscopic esophagitis after LSG in patients who were converted to LRYGBP

Endoscopic Esophagitis (n=38)					
	Grade A (n=15)	Grade B (n=13)	Grade C (n=5)	Barrett´s esophagus (n=5)	p
Manometry					
LESP (mmHg)*	6,82+1,76	6,40+3,24	6,41+ 1,53	5,07+2,26	0,58
Length (cm)					
Total	3,7+0,6	3,4+0,5	3,3+0,5	3,5+0,6	
Abdominal	0,4+0,0	0,0	0,0	0,0	
24 h pH monitoring					
% time pH <4	8,72+5,24	15,6+3,0	18,54 +1,53	17,58+6,8	0,001
DeMeester score	55,25+10,61	52,6+3,69	65,40+11,632	88,20+38,09	0,036

Regarding acid reflux test, pathologic increased acid reflux was observed in all patients submitted to this evaluation. The %time of pH<4 were significant elevated in patients with esophagitis grade B, C and Barrett´s esophagus compared with esophagitis grade A (p=0.001), stablishing an Honest Statistical Difference (HSD) of 6.63. The DeMeester´s score was more elevated in patients with grade C esophagitis and Barrett´s esophagus (65.40+15.2 and 88.20+38.8 respectively) and showed significant difference (p=0.036) compared with esophagitis grade A and B, stablishing HSD of 32.36.These results, in addition to the presence of symptoms and the endoscopic findings, supported the indication for conversion to gastric bypass.

**FIGURE 3:**
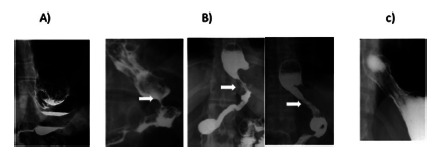
Radiologic confirmation of hiatal hernia post-sleeve gastrectomy: A) hiatal hernia with intramediastinal fundus; B) hiatal hernia with mesogastric stricture; C) dilated cardia with residual fundus

**FIGURE 4:**
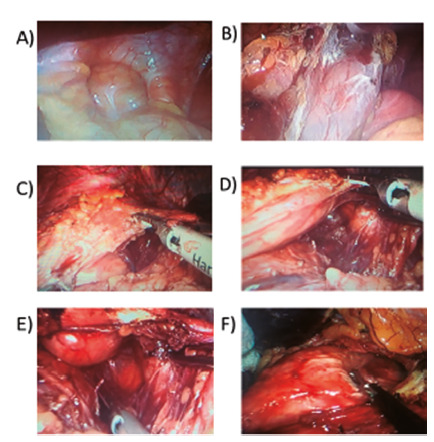
Intraoperative confirmation of hiatal hernia after sleeve gastrectomy, showing: A) dilated hiatus; B) anterior gastric fundus dissection; C) intramediastinal gastric fundus dissection; D) gastric fundus pulled down after intramediastinal dissection; E) posterior view of herniated gastric fundus; F) hiatal hernia with intramediastinal gastric fundus

Radiologic abnormalities of sleeve were observed in 35 patients. Cardia dilatation was observed in 18 patients and hiatal hernia in 11. Few patients presented middle gastric stricture (n=6, two of them associated with twist or angulation of sleeve, Table 4). In the cardia dilatation group was not detected Barrett´s esophagus probably because these patients were operated upon very early due esophagitis before the development of Barrett´s metaplasia. Figure 3 shows the radiological abnormalities found in patients operated several years before, confirmed during the reoperation (Figure 4).

**TABLE 4:** Endoscopic findings and radiological abnormities after LSG in patients who were converted to LRYGBP

		Radiological evaluation			
		Normal	Dilated	Hiatal	Mesogastric
		Sleeve	Cardia	Hernia	Stricture
Endoscopic findings	n	n	n	n	n
Esofagitis					
A	15	2	10	3	
B	13	1	5	6	1
C	5			2	3
Barrett´s esophagus	5		3		2
Total	38	3	18^	11*	6

*Presence of hiatal hernia comparing Barrett vs. no Barrett´s patients (p=0,29); ^=presence of cardia dilatation comparing Barrett vs. no Barrett´s patients (p=0,65

The LES characteristics and radiological findings after sleeve gastrectomy are shown in Table 5. Incompetent LES was found either after LSG with or without dilated cardia or hiatal hernia. 

This finding revealed that gastroesophageal reflux after sleeve gastrectomy is mainly due to progressive dilatation of cardia or the presence of hiatal hernia along time after surgery.

**TABLE 5:** Manometry, 24 h pH monitoring, and radiological evaluation after LSG in patients who were converted to LGBP

	Radiological evaluation			
	Normal	Dilated	Hiatal	Mesogastric
	Sleeve	Cardia	Hernia	Stricture
	(n=3)	(n=18	(n=11)	(n=6)
Manometry				
LESP (mmHg)	5.5+2.1	6.4+1.6	6.8+2.9	4.1+2.6
Total lenght (cm)	3.4+0.7	4.0+0.6	3.0+0.9	3.5+0.0
Abdominal length (cm)	0	0	0	0
24 h pH * monitoring				
% time pH<4	8.3+0.2	15.5+10.1	18.4+5.5	17.6+8.4
DeMeester score	22.4+2.5	31.8+32.6	65.4+39.6	89.4+3.7

*=%time of pH<4 in patients with normal sleeve vs. dilated cardia, hiatal hernia or mesogastric stricture patients (p=0.04, IC 95%, 0.11-23.77; De Meester´score in patients with normal sleeve vs. dilated cardia, hiatal hernia or mesogastric stricture patients (p=0.03, IC 95%, 6.26-102.97)

## DISCUSSION

GER is the very frequent after LSG promoting appearance of “de novo” reflux symptoms, esophagitis and even Barrett´s esophagus^3^ ^,^ ^8^ ^,^ ^10^ ^,^ ^23^ ^,^ ^24^ ^,^ ^26^. It seems that our results are more alarming in terms of the appearance of symptoms, esophagitis or Barrett’s post LSG, but these findings are very consistent with the published data^10^ ^,^ ^23^ ^,^ ^26^.

In recent years, a vast amount of literature concerning the conversion of LSG to LRYGBP has been published and GER has been the main cause of conversion. In some reports patients who underwent conversion to LRYGBP following primary LSG, 12-50% were converted due to GERD. However, there is no consensus about the incidence of GER after LSG because the surgical technique is difficult to be standardized and therefore, the results are different^1,6,15, 17,22,24,29^. For other authors, GER was the cause of conversion in only 4%. GER after LSG is due to multifactorial mechanisms such as decreased LES pressure, disruption of the sling fibers, modification of the esophagogastric angle, elevated intra-gastric pressure and ineffective esophageal peristalsis affecting the esophageal clearance ^1^ ^,^ ^10^ ^,^ ^12^ ^,^ ^16^ ^,^ ^19^ ^,^ ^21^ ^,^ ^24^ ^,^ ^25^. In high-volume centers, where strict criteria for patient selection for LSG are applied, the expected incidence of reoperations for “de novo” or persistent severe GER and patients not responding to medical treatment is low^4^ ^,^ ^6^ ^,^ ^10^ ^,^ ^15^ ^,^ ^16^ ^,^ ^17^ ^,^ ^22^ ^,^ ^23^ ^,^ ^26^ ^,^ ^29^. The mean period for conversion was approximately 3-5 years, very similar to our experience. The average amount of time that reflux symptoms appeared and the moment of conversion to LRYGBP varied greatly. The mean interval between the two procedures could be very early after operation, but the mean interval between the two procedures oscillated from 26 to 33 ± 27.8 months for severe GERD (2-60)^1^ ^,^ ^3^ ^,^ ^11^ ^-^ ^16^. The published rate of complications after conversion range from 16.7-31%, mainly grade II or IIIa (Clavien-Dindo´s classification), with conversion rate to open procedure in 7.5% without mortality. Our results are very similar^2^ ^,^ ^16^ ^,^ ^24^.

 Most publications concerning conversion to LRYGBP after LSG have evaluated only symptoms and few analyzed the endoscopic findings in patients that have been converted to LRYGBP. In this study, in patients who developed GERD after LSG, presented different grades of erosive esophagitis, hiatal hernia and Barrett´s esophagus. Stricture is other anatomic and pathophysiologic factor involved in the appearance of “de novo” reflux symptoms because in this condition high intragastric pressure should occur. In our experience in agreement with other authors, few patients presented gastric strictures (8.3% to 12%)^1^ ^,^ ^9^ ^,^ ^18^ ^,^ ^22^ ^,^ ^27^ ^,^ ^30^.

The outcome after conversion has been very successful in all the publications: 83-100% of patients resolved or improved their symptoms and 75-80% patients were able to stop their antacid medication^3^ ^,^ ^4^ ^,^ ^10^ ^,^ ^11^ ^,^ ^13^ ^,^ ^15^ ^,^ ^18^ ^,^ ^24^. Strictures improved obviously in 100% of patients, successful hiatal repair obtained in 50%, 80% showed remission of Barrett´s esophagus and patients had score 5.1 on average in the BAROS scale which denotes a very good outcome after conversion to LRYGBP^6^ ^,^ ^9^ ^,^ ^22^. 

Few authors have evaluated objectively the functional aspects in order to decide conversion to LRYGBP. It could be argued that in patients with clear reflux symptoms or esophagitis post-SLG, it would not be necessary to carry out these studies since it is recognized and accepted that these patients should be converted to GBP. The reasons for carrying them out are: 1) it provides valuable necessary information considering the possible good or poor response to PPI´s treatment and its continuity for long time; 2) there is a group of severe symptomatic patients who do not present esophagitis (non-erosive reflux disease ) and therefore they should be studied in particular with manometry, 24 h pH monitoring, even with scintigraphic bile reflux assessment; and 3) in the same sense, it is necessary to specify the severity of the reflux to determine the long-term prognosis.

In this study manometry showed defective LES function and pathologic pH monitoring in all patients. El Chaar et al.^8^ mentioned that 50% of converted patients to LRYGBP were symptomatic with poor response to PPI´s who presented pathologic 24 h pH monitoring. Felsenreich et al.^9^ reported decreased of acid exposure time from 36.8 to 3.8% and the mean DeMeester score from 110.0 to 16.3, respectively. 

Additionally, Hawasli et al.^13^, reported the experience with placement of LINX^®^ system in 13 patients submitted previously to SG as an alternative to LRYGBP conversion in managing refractory post-LSG reflux. Bravo score for reflux was 46+26 before the procedure. In one patient, the system required removal of the LINX^®^ due to severe dysphagia on the 18^th^ postoperative day. However, large studies are required to assess its safety and long-term efficacy.

Other options for surgical treatment to treat reflux after sleeve have been published such us to add fundoplication (Nissen sleeve or Dor sleeve) or bipartition, but up to now these procedures are even in evaluation.

The limitations of this study were its descriptive design and few patients included. In the other hand, the strengths were: 1) there are few publications that reported objective evaluation before the conversion to LRYGBP as this one did (the majority of papers report only symptomatic or endoscopic evaluations before the second operation); 2) it is a prospective cohort; and 3) the results are useful for validate the indication for conversion. 

## CONCLUSION

Patients with reflux symptoms and esophagitis or Barrett´s esophagus after SG present defective lower esophageal sphincter function and increased acid reflux. These conditions support the indication of conversion to LRYGBP.
